# Virtual screening of gene expression regulatory sites in non-coding regions of the infectious salmon anemia virus

**DOI:** 10.1186/1756-0500-7-477

**Published:** 2014-07-28

**Authors:** Álvaro Díaz, Katherine García, Andrea Navarrete, Gastón Higuera, Jaime Romero

**Affiliations:** 1 Instituto de Nutrición y Tecnología de los Alimentos. INTA, Universidad de Chile, Avenida El Líbano #5524, Macul, Santiago, Chile; 2 Programa de Doctorado en Acuicultura, Programa Cooperativo Universidad de Chile, Universidad Católica del Norte, Pontificia Universidad Católica de Valparaíso, Antofagasta, Chile

**Keywords:** Non coding region, Infectious salmon anemia virus, Virulence

## Abstract

**Background:**

Members of the *Orthomyxoviridae* family, which contains an important fish pathogen called the infectious salmon anemia virus (ISAV), have a genome consisting of eight segments of single-stranded RNA that encode different viral proteins. Each of these segments is flanked by non-coding regions (NCRs). In other *Orthomyxoviruses*, sequences have been shown within these NCRs that regulate gene expression and virulence; however, only the sequences of these regions are known in ISAV, and a biological role has not yet been attributed to these regions. This study aims to determine possible functions of the NCRs of ISAV.

**Results:**

The results suggested an association between the molecular architecture of NCR regions and their role in the viral life cycle. The available NCR sequences from ISAV isolates were compiled, alignments were performed to obtain a consensus sequence, and conserved regions were identified in this consensus sequence. To determine the molecular structure adopted by these NCRs, various bioinformatics tools, including RNAfold, RNAstructure, Sfold, and Mfold, were used. This hypothetical structure, together with a comparison with influenza, yielded reliable secondary structure models that lead to the identification of conserved nucleotide positions on an intergenus level. These models determined which nucleotide positions are involved in the recognition of the vRNA/cRNA by RNA-dependent RNA polymerase (RdRp) or mRNA by the ribosome.

**Conclusions:**

The information obtained in this work allowed the proposal of previously unknown sites that are involved in the regulation of different stages of the viral cycle, leading to the identification of new viral targets that may assist future antiviral strategies.

## Background

The infectious salmon anemia virus (ISAV) is a member of the *Orthomyxoviridae* family and the genus *Isavirus*[1,2]. The infectious cycle of these viruses involves the synthesis of three types of RNA that are associated with different stages of infection in the host cell. The viral RNA (vRNA) genome is found in the virion. The vRNA is released into the cytoplasm of the infected cell and transported to the nucleus for viral transcription and replication. During transcription, the vRNA serves as a template for the synthesis of messenger RNA (mRNA), which is necessary for the production of viral proteins. In turn, the identical vRNA is replicated to generate copies of the genomic segments by synthesis of complementary positive polarity RNA (cRNA). The cRNA is used as a template for the generation of new genomes for future viral progeny [1]. Open reading frames (ORFs) on each genomic segment are flanked on their terminal ends by non-coding regions (NCRs). These NCRs are related to the regulation of the viral infectious cycle [3-6].

The NCRs contain conserved sequences that vary in length among the various genera of the family *Orthomyxoviridae*[3,7]. The first 12 and 13 nucleotides have been reported to correspond to conserved sequences in the 3′ and 5′ ends, respectively, of all segments of the influenza A vRNA [7,8]. Structurally, these conserved sequences in influenza A have been described as partially complementary and capable of interacting in *cis* within each segment of RNA. This interaction forms structures called panhandles [5,9]. In *Orthomyxoviruses*, transcription of the genome requires vRNA to act as a template for each genomic segment; for transcription to occur, the conformation adopted via the folding of the NCR is essential [9].

Despite technological advances, experimentally determining the exact secondary structure adopted by RNA is difficult [10]. Therefore, generating an accurate prediction of the structures that RNAs will form is relevant in the understanding of the relationship between their structure and function. In *Orthomyxoviruses*, the folding of the NCR determines its function during the viral life cycle, as has been reported for influenza A, B, and C and for thogotovirus [3,9,11-14]. Within the NCR, nucleotides are involved in the recognition of the viral RNA-dependent RNA polymerase (RdRp) and signals responsible for the termination of transcription and polyadenylation [15,16]. Therefore, these regions are vital to the replication and transcription of the viral genome [17].

Other studies have attributed a regulatory role in viral protein translation to the NCRs based on the observation that the 5′ NCR of the influenza viral mRNA contains critical determinants that ensure selective translation over cellular mRNAs [18]. Specific sequences within the NCRs have been shown to be involved in virulence, resulting in the NCRs being proposed as candidate modulators of pathogenicity [19].

Limited knowledge is available for the NCRs in ISAV, and few sequences for these elements from ISAV have been reported [4,5,20,21]. However, the availability of these sequences would allow an increase in the depth of knowledge on the structure and function of the NCRs of ISAV, using the influenza virus as a model. Therefore, this study aims to determine possible functions of the NCRs of ISAV by proposing an association between their molecular architecture and their role in the viral life cycle. Thus, available non-coding sequences for ISAV isolates were collected, alignments were performed to obtain a consensus sequence of NCRs within the genus Isavirus, and conserved regions were determined in these sequences by comparing ISAV with other *Orthomyxoviruses*. Secondary structure modeling of the NCRs from each isolate was then conducted, and possible structural motifs related to regulation of gene expression in ISAV were identified.

## Results

### Analysis of non-coding regions in ISAV

**Figure 1:**
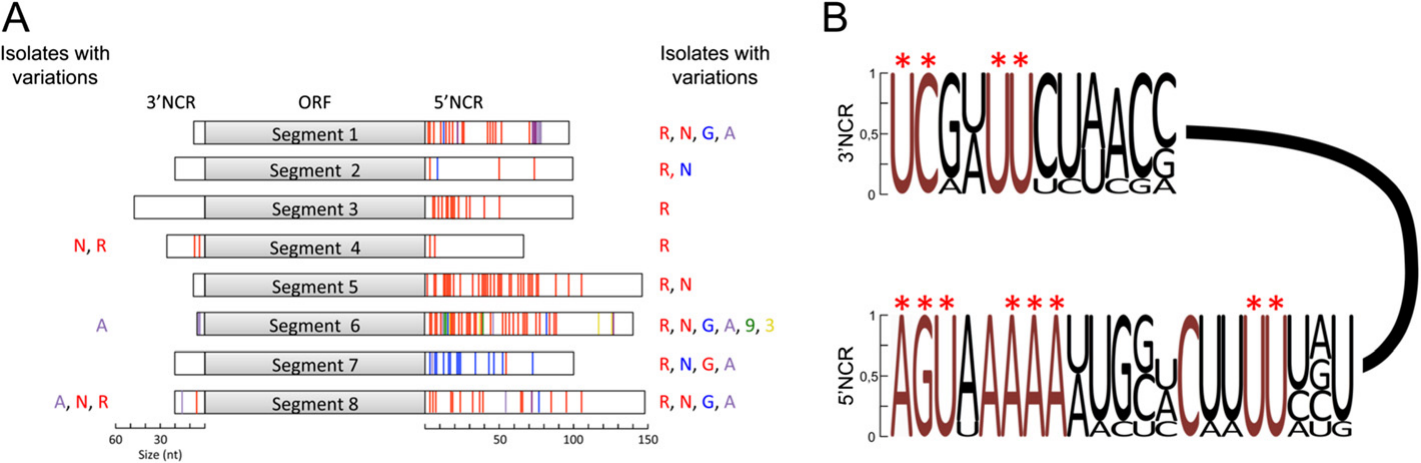
**Comparative analysis of the NCR of ISAV. A**. Differences in length and sequence among the NCRs of the 8 genomic segments of ISAV. The positions showing variation are represented by vertical colored lines. The color corresponds to the isolates that present such variation in each NCR. Isolate AD, A; Isolate NB, N; Isolate 39, 3, Isolate 98, 9; Isolate GL, G, and Isolate RP, R. **B**. Consensus sequence of the terminal regions of each NCR. Proportion of each nucleotide is indicated by the font size. Red letters indicate the nucleotides conserved among the isolates studied (intragenus conservation). Red asterisks indicate nucleotides conserved with influenza A (intergenus conservation). Coding region is represented by solid black line.

An alignment of the non-coding vRNA derived from six different ISAV isolates (AD, NB, 39, 98, GL, and RP) was performed, and from this alignment, a comparison of the NCRs at the ends of each of the segments of the isolates was performed. Figure 1A illustrates the eight segments of ISAV in schematic form and represents the different lengths and sequence variations in the NCRs of the analyzed isolates. The lengths and sequences of each of the 3′ NCR of genome segments 1, 2, 3, 5, and 7 were identical among all isolates studied. By contrast, segments 4, 6, and 8 presented sequence differences that are represented in Figure 1A as vertical colored lines, in which each color indicates the isolate that contains this variation. Notably, isolates NB and RP (N and R in red) displayed differences in the identical positions in segments 4 and 8 (Figure 1A). Furthermore, the 5′ ends of the NCR were larger (from 67 to 148 nucleotides) and more variable compared to the 3′ NCR (with extensions between 8 and 48 nucleotides long). Similar to what was observed in the 3′ region, isolates NB and RP presented variations in the 5′ region at the identical nucleotide positions in segments 1, 5, 6, and 8 (Figure 1A).

### Identification of conserved nucleotides and consensus sequence of the NCRs of ISAV

Based on the alignments described above (Additional file 1: Figure S1), conserved regions at the termini of each NCR in the viral genome were identified. Consensus sequences for the 3′ and 5′ NCRs of the genomic segments of ISAV were then generated. These consensus sequences are presented graphically in Figure 1B, in which the proportion of each nucleotide base is indicated by the font size. Within a consensus sequence, positions were identified that contained 100% identity between the different isolates of ISAV (intragenus conservation, Figure 1B, red letters). The consensus sequence at the 3′ end of the NCR comprised 12 nucleotides and had high conservation until position 8 (Figure 1B), and segment 5 was the only segment that presented differences from this sequence (Additional file 1: Figure S1). Furthermore, the consensus sequence of the 5′ NCR contained 21 nucleotides. The terminal 8 nucleotides presented 100% intragenus conservation (red letters), except for position 4, which had an A→U variation in segment 3 (Figure 1B). Downstream, positions 17 and 18 were conserved at the intragenus level and corresponded to a location described by other authors [4,20,21] as a possible polyadenylation signal (U *stretch*) (Figure 1B). To examine which nucleotides were conserved at the intergenus level, the consensus sequences obtained from both NCRs of ISAV were compared with the information available for NCRs in influenza A [14,22]. At the 3′ end of the NCR, the positions described as being conserved at the intragenus level were also conserved at the intergenus level (Figure 1B, asterisk above each nucleotide position). At the end of the 5′ NCR, of the 10 nucleotides that were conserved at the intragenus level, 8 remained conserved at the intergenus level in comparison with influenza (Figure 1B, asterisk above each nucleotide position).

### Description of gene expression regulatory elements

#### Structural motifs in vRNA involved in viral transcription and in cRNA involved in viral replication

**Figure 2:**
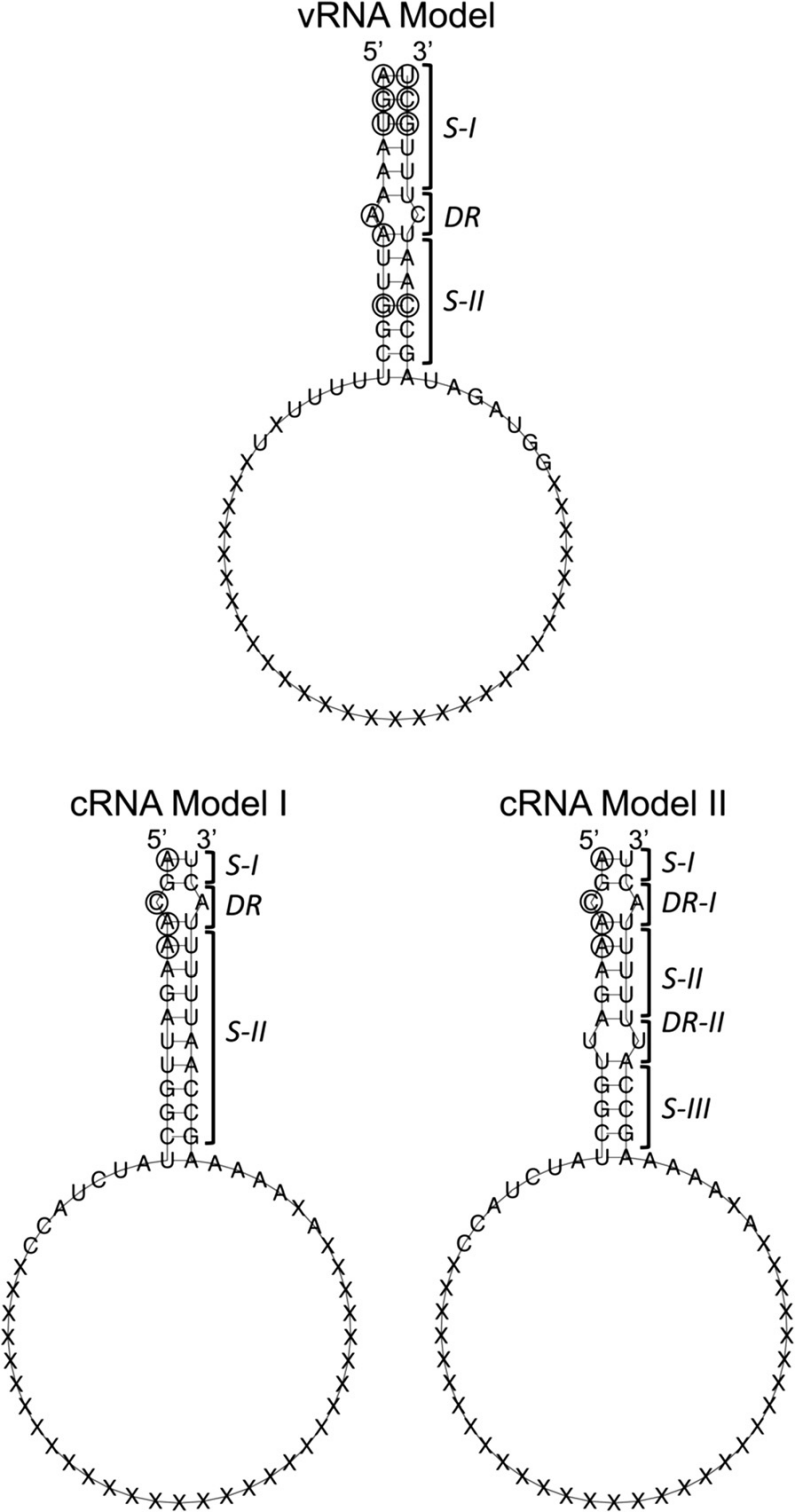
**General folding models for the vRNA and cRNA of ISAV.** The interaction between the 3′ and 5′ ends of both the vRNA and cRNA generates stable secondary structures with characteristic domains. S-I: stem I; S-II: stem II; S-III: stem III; DR (I or II): denatured region (I or II). The ORFs of each segment are represented by “X”.

In the orthomyxoviruses, transcription of the genome requires vRNA from each genomic segment to serve as a template. For this process to occur, the conformation adopted by the folding of the NCR is essential [9]. To determine the secondary structure adopted by the NCR of ISAV, 2D predictions were generated for each genomic segment of the studied isolates. The folds obtained for the different vRNA segments generated common structural motifs and followed a stem-loop-stem pattern in the majority of cases (Additional file 2: Figure S2). To facilitate the visualization of these common motifs in a single structure, a model was generated using the consensus sequence of the NCR in the vRNA (Figure 2). In this model, a conserved and stable terminal double-stranded region called *Stem I* was observed, followed by a region called *Denatured Region* and, a double-stranded region called *Stem II*, which varied in length between the segments (Additional file 2: Figure S2).

These structural motifs observed in ISAV are analogous to those described in the panhandle structure of influenza [8,9,15]. The formation of this type of structure in the NCRs of influenza is crucial, as these structures contain the nucleotides that form the binding site for the RdRp. The nucleotide positions involved in the recognition of viral RdRp in the 5′ NCR of influenza correspond to A^1^, G^2^, U^3^, A^7^, A^8^, C^9^, A^10^, A^11^, and G^12^[17] and in the 3′ NCR correspond to U^1^, C^2^, G^3^, and C^11^[9,15,17]. Of this set of nucleotides described in influenza, six were found to be conserved in the 5′ NCR of ISAV, and four were conserved in the 3′ NCR of ISAV (Figure 2, positions marked by circles in the vRNA Model). In both NCRs, the nucleotides potentially involved in the recognition of the RdRp were located in the *Stem I* and *Stem II* motifs and in the *Denatured Region*. These nucleotides are highlighted with circles in Figure 2.

Replication of the viral genome involves the conversion of cRNA to vRNA. Therefore, we also modeled the secondary structure formed between the terminal ends of the cRNA to determine which nucleotides may be involved in genome replication. In contrast to the general model observed for the vRNA, the folding observed for each segment of the cRNA (Additional file 3: Figure S3) can be grouped into two consensus secondary structure models (Figure 2, cRNA Model I and II). Model I contained the identical structural motifs observed in the vRNA, and these motifs were similarly organized. Model II also maintained the identical organization as observed in the vRNA; however, a stem structure of greater length was interrupted by a *Denatured Region II* to generate stem structures II and III in this model. After a comparison with influenza, as was done for the vRNA, candidate cRNAs were not identified that interact with the RdRp in the 3′ NCR. By contrast, in the 5′ NCR, positions A^1^, C^3^, A^4^, and A^5^ were identified as candidates for interacting with the RdRp (Figure 2, positions marked with circles in the cRNA Model I and II). These nucleotides are located in positions that are highly conserved in comparison with influenza A [15]. This is the first identification of a putative RdRp binding site in ISAV.

To validate our observations, the 2D models obtained in this study for segment 8 (Additional file 2: Figures S2 and Additional file 3: Figure S3) were compared with the experimental results obtained by Brinson and colleagues [5]. Our bioinformatics analysis showed that the secondary structure model obtained for segment 8 had identical regions to those described experimentally and even predicted the non-canonical U-G interactions previously detected by NMR experiments.

#### Elements regulating viral translation

The efficiency of translation initiation is determined, in part, by a specific combination of nucleotides that surround the AUG, known as the Kozak consensus sequence (GCC^A^/_G_CC**AUG**G). The A at position -3 and the G at +4 (with the A of the AUG being at position +1) correspond to the critical nucleotides that determine the strength of translational initiation [23,24]. The remaining noncritical nucleotides of the Kozak sequence have a remarkable effect only when one of the critical nucleotides is absent [24]. In this study, an arbitrary score of the AUG strength was assigned according to the presence or absence of the two critical nucleotides for efficient translation initiation (weak: both absent; strong: one was present; optimal: both were present). Intermediate values were also included in this score based on the presence of non-critical nucleotides in the Kozak consensus sequence from 0 to 5, with zero being the absence of such nucleotides in the Kozak sequence and 5 being the presence of all such nucleotides. Using these data, a score was assigned for the strength of the context of AUGs in the genome segments of the studied isolates. Based on this ranking, a Kozak sequence was identified in all segments of ISAV, and all segments, except for segment 8, presented an optimal sequence context (Table 1). Segment 8 was a special case, as it encodes two polypeptides originating from two different ORFs. Of these two ORFs, the context of the AUG for the second ORF for all isolates was optimal. By contrast, the context of the AUG for the first ORF was strong, with variations of the intermediate values among the isolates. For NB and RP, a strong +2 context was observed; for the remainder of the isolates, the AUG context was a strong +3 (Table 1). Based on this information, NB and RP, which have a “weaker” context for translational initiation at this ORF than the rest of the isolates studied, should initiate the synthesis of the protein of the first ORF less efficiently.

**Table 1:** Strength of context surrounding the AUG codons (Kozak sequence) in the mRNA of each segment of ISAV

**Segment**	**AUG context**	**Strenght**
1	CUA**A**GA*AUG***G**	Optimal + 0
2	AUA**A** ***CC*** *AUG***G**	Optimal + 2
3	UAA**G**AG*AUG***G**	Optimal + 0
4	UU ***C*** **A**AG*AUG***G**	Optimal + 1
5	UUA**A**AG*AUG***G**	Optimal + 0
6	***GC*** A**A**AG*AUG***G**	Optimal + 2
7	U ***C*** U**A** ***C*** A*AUG***G**	Optimal + 2
8 ORF 1 (R, N)	UUU**A** ***CC*** *AUG*A	Strong + 2
8 ORF 1 (3, 9, A, G)	U ***C*** U**A** ***CC*** *AUG*A	Strong + 3
8 ORF 2	AU ***C*** **A** ***C*** A*AUG***G**	Optimal + 2

Segments 1 to 7 have an optimal sequence for the initiation of protein synthesis; differences were observed only in the number of non-critical nucleotides. ORF 1 of segment 8 has a strong sequence for the initiation of protein synthesis and has 2 variants. The presence of nucleotides matching the Kozak consensus is indicated as follows: bold letters, critical nucleotides; bold-italics, non-critical nucleotides; italics, AUG codon.

#### Regulatory elements involved in transcription termination

A uracil-rich region called the U stretch, which functions as a signal for polyadenylation and transcription termination, has been described in influenza A [15]. Three conditions must be met for this sequence to be functional: it must possess 5–7 uracil residues, it must be located 16 nucleotides from the 5′ terminus of the vRNA, and a duplex RNA secondary structure adjacent to the U stretch must form [16]. When searching for these elements in ISAV, U-stretch regions in each viral segment in the different isolates were identified (Table 2). When comparing these regions with the region reported for influenza, the U stretch in Isavirus was shorter. The sequence consisted of 3–5 U residues, with 4 uracils being the most frequent length, and was located 13–15 nucleotides from the 5′ terminus of the vRNA (Table 2). The formation of secondary RNA structures also occurred adjacent to the U stretch (Figures 2, Additional file 2: Figure S2, and Additional file 3: Figure S3). Despite the reported differences of these regions with those from influenza, a number of patterns were identified that remain constant between the different segments of the different isolates of ISAV. These findings facilitate the identification of this signal in the study of new viral isolates.

**Table 2:** Characterization of the U stretch region in the 3′ NCR of the vRNA

**Segment**	**U residues**	**Distance from 5′ end of vRNA**
1	4	14
2	4	14
3	4	13
4	5	13
5	3	15
6	4 - 5	14
7	4	14
8	4	13

For each segment, the number of residues comprising the U stretch and the distance from the terminal nucleotide of the 5′ NCR are reported.

## Discussion

Previous studies have suggested that ISAV uses mechanisms similar to those of other members of the family *Orthomyxoviridae* to perform molecular processes in the viral life cycle [2,4,5,25]. In these viruses, the interaction of the terminal regions of the NCRs and the subsequent formation of panhandle structures [5] are key in performing these processes.

The analysis of the ISAV isolates included in this study revealed that the NCRs possess highly conserved areas, both among segments of the same isolate and among isolates (intragenus). This feature has also been described in influenza, whose terminal NCR ends have low divergences and a slower speed of evolution compared to the ORFs of each genomic segment [14]. This phenomenon is rational from a functional point of view, as the promoters for replication and transcription are found in these regions in several genera of the family *Orthomyxoviridae*[3,8,13,26]. These observations support the hypothesis that the NCRs also contain viral cycle regulatory sequences in ISAV.

In this work, conserved nucleotide positions in ISAV were identified that are homologous to nucleotides that have been described as candidates for interaction with the RdRp in influenza (Figure 2). This information is particularly relevant when analyzed in conjunction with the predicted folding model because, in addition to the identities of nucleotides, the viral polymerase is also able to recognize the molecular architecture in which nucleotides are immersed [15]. In ISAV, complementarity between the 3′ and 5′ ends of the NCRs of the genomic segments has been theoretically shown [4,5,20]. However, only the Brinson group [5] has experimentally confirmed the interaction of the NCR in segment 8 by nuclear magnetic resonance (NMR) analysis and thermal melting data. This group identified pairing between nucleotides present in the 3′ and 5′ ends of termini of the vRNA and the cRNA. To verify the results obtained in this study, the 2D models obtained for segment 8 (Additional file 2: Figures S2 and Additional file 3: Figure S3) were compared with the experimental result obtained by Brinson and colleagues (2011). Our bioinformatics analysis predicted a secondary structure for segment 8 that contained the identical regions as those described experimentally. Moreover, non-canonical U-G interactions were formed in our model that was also detected in the NMR experiments. The agreement obtained between our data and the experimental results serves to support the models predicted with the methodology utilized in this report.

Our 2D modeling shows that the location of the nucleotides contacting the viral polymerase and the molecular architecture of this contact region are similar to those described for influenza A [17]. In influenza, the less stable loop and stem regions have been described to be favorably recognized by the polymerase [10,15]. In our proposed model for ISAV, stem I is conserved in both the vRNA and the cRNA in all isolates; thus, influenza likely forms a similar structure. In addition, an adenine is at the 5′ terminus of the vRNA and in the complementary cRNA. The presence of the adenine at that position has been reported in influenza, in which this nucleotide has been shown to be involved in the initiation of the synthesis of vRNA and cRNA [15].

**Figure 3:**
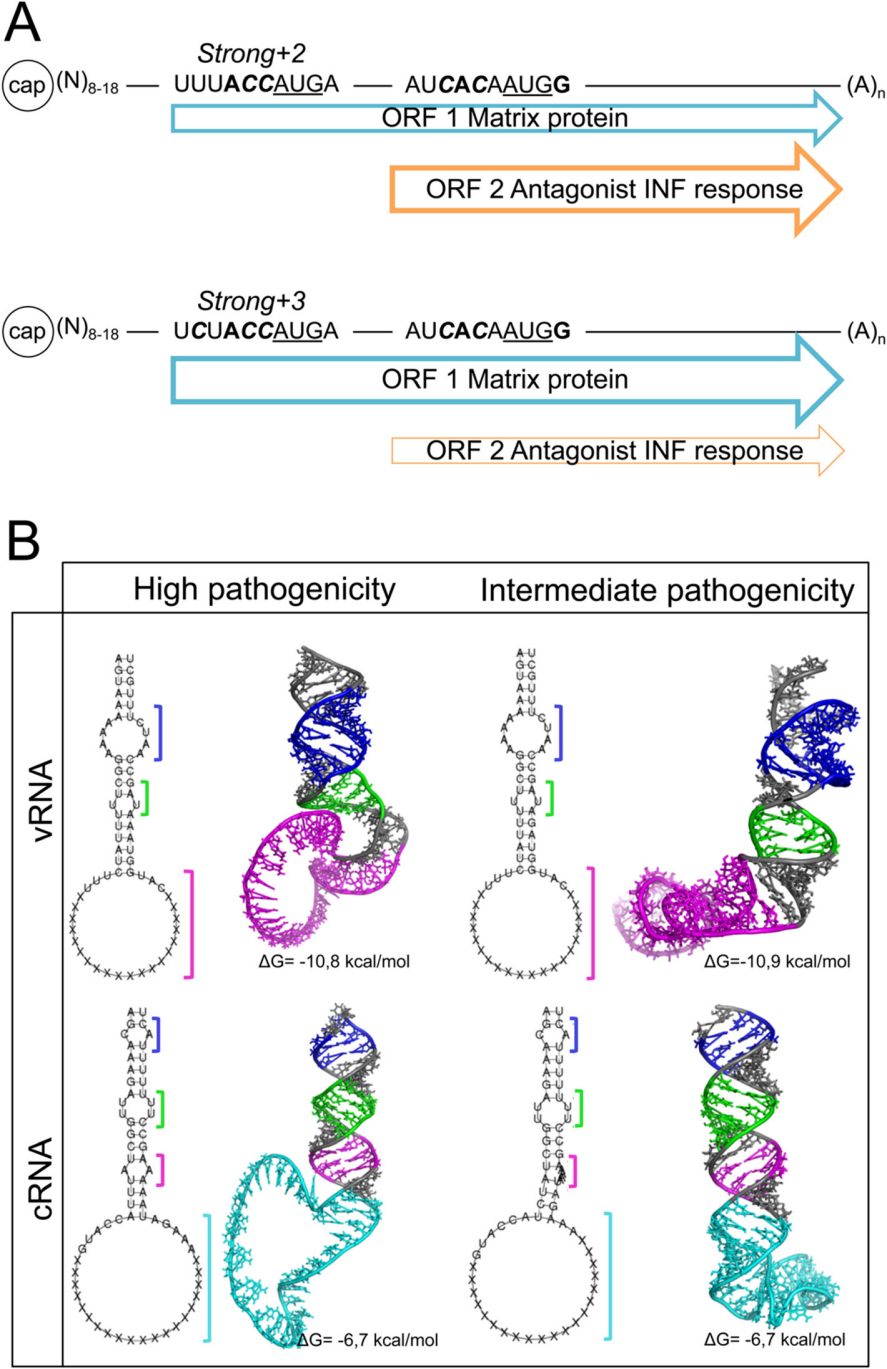
**Schematic model of gene regulation and three-dimensional structure prediction of the NCR of segment 8 of ISAV. A**. The potential leaky scanning mechanism for gene expression of the second ORF in segment 8 of ISAV. The mRNA transcripts for segment 8 for two groups of isolates studied that differ in their Kozak sequence (see Table 1). Start codon are underscored. Critical nucleotides are in bold. Non-critical nucleotides are in bold and italics. **B**. Three-dimensional modeling of the folding of the vRNA and cRNA calculated at 15°C for intermediate- and high-pathogenicity isolates and the corresponding free energy (ΔG). The theoretical spatial distribution of the domains described in the 2D models is highlighted in color (in vRNA, purple and green represent denatured regions, and the ORF is represented in pink; in cRNA, purple, green, and pink represent the denatured regions, and the ORF is presented in light blue; in both models, the stems are represented in gray).

Differences in sequences observed between the NCRs from ISAV isolates determine the folding of the terminal ends of the genome segments. However, these variations may also affect regulatory sequences that are involved in key processes during the viral life cycle [19]. This phenomenon occurs in segment 8, as several of the nucleotide variations in this segment affect the Kozak sequence that surrounds the AUG of the mRNA. The efficiency of translation initiation is largely determined by three features present in the mRNA: the 5′ cap, the context surrounding the AUG (Kozak sequence), and the position of the AUG (if there is more than one) [24]. In each segment 1 to 7, translation of the ORF originates from a single AUG codon that has a Kozak sequence identical among each of the segments in all of the isolates. By contrast, segment 8 is bicistronic, and the AUG sequence context of segment 8 differs among the isolates studied in this work. Based on the criteria that influence the efficiency of translation initiation, the first ORF of the NB and RP isolates should be translated less efficiently compared to the other isolates studied (Strong +2, Figure 3A). This decrease in translation efficiency in these isolates would encourage the translation of the second ORF because the RdRp bypasses the suboptimal codon, resulting in a process known as “leaky scanning”. This process has been described as one of the mechanisms used by viruses to modulate protein translation [24]. The protein encoded by the second ORF of segment 8 in ISAV has been described as a structural protein that is capable of facilitating the hybridization of ssRNA and dsRNA and antagonizing the type I IFN response. This protein is homologous to NS1 of influenza; the primary role of NS1 is the inhibition of the host immune response and is related directly to the virulence of the virus [27].

In influenza A and B [19], the NCRs have been shown to have a modulatory role in virulence. Subsequently, studies have shown a relationship between the sequence of the NCRs and the pathogenesis of various influenza viral isolates, highlighting specific nucleotide differences located within the consensus sequence that are present only in highly pathogenic isolates [28]. Based on the virulence of the different isolates used in this study (Table 3), the observed NCR differences at the genomic level (Figure 1A and Additional file 1: Figure S1) were related to the ability to cause disease in the host. With this information, we proposed a role for the NCRs as potential modulators of pathogenicity. The NB and RP isolates (NA genotype) are the most pathogenic isolates evaluated in this study, and they are grouped together at the nucleotide sequence and structural levels based on both NCRs. The nucleotide differences present in these isolates relative to the other isolates are observed primarily between the U stretch and the stop codon of each ORF in the 5′ NCR of vRNA. These differences do not form part of the folding between the NCR of each viral segment and, therefore, would affect viral replication on their own [12].

**Table 3:** Pathogenicity of the ISAV isolates used in this study

**ISAV strain**	**Pathogenicity in** ***S. salar***	**Experimental data**	**Reference**
RPC/NB 98-049-1	High	100% cumulated mortality	[29-31]
NBISA01	High	95% cumulated mortality	[29-32]
390/98	Intermediate	79% cumulated mortality	[29-31,33]
Glesvaer/2/90	Intermediate	67,5% cumulated mortality	[29,33,34]
ADL-PM 3205 ISAV07	Intermediate	Chilean isolated HPR-5	[20]
982/08	Unknown	---	[21]

The relevant experimental data for determining the order of pathogenicity is reported with respective references.

**Figure 4:**
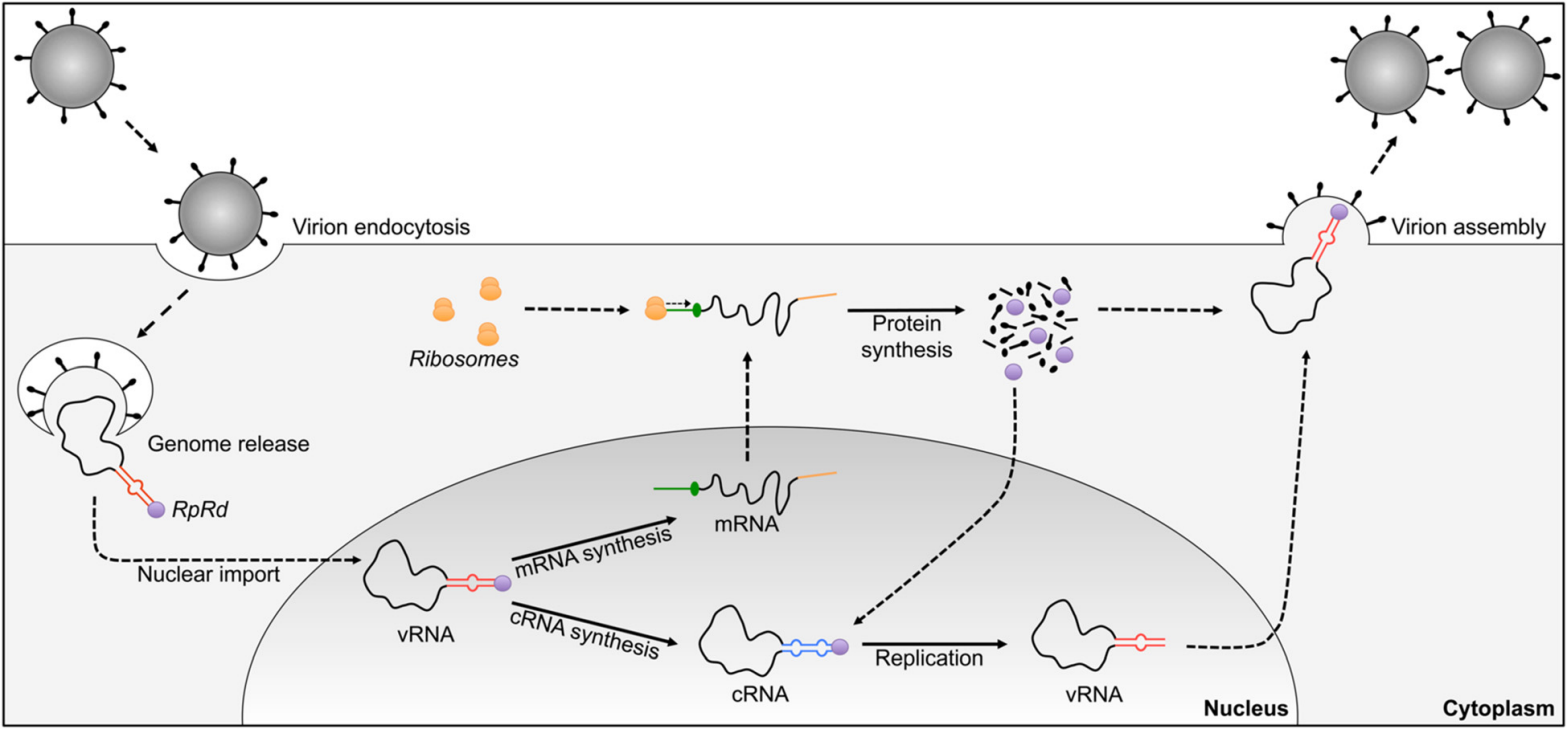
**Simplified diagram of the stages of the viral cycle regulated by the folding of the NCR of ISAV.** For easy viewing, the ISAV genome is represented as a single genomic segment. In the nucleus, the viral genome (vRNA) is transcribed by the RdRp (purple circle) bound to each genomic segment to generate mRNA and cRNA. In the cytoplasm, the ribosomes recognize and scan the 5′ NCR region of the mRNA until the AUG codon to initiate the synthesis of viral proteins, which includes the RdRp necessary for genome replication (synthesis of vRNA) and of the rest of the viral proteins involved in the assembly of the mature viral particle. Continuous lines: stages regulated by NCR; dotted lines: the remaining stages of the viral cycle. The folds adopted by the NCR of vRNA (red line) and cRNA (blue line) are highlighted; in mRNA, the 5′ NCR is shown (green line), including the AUG codons (green circle), and the 3′ NCR is shown, including the poly-A tail (yellow line).

Furthermore, the 3D model constructed for the folding of the NCR of segment 8 indicates that the panhandles in the vRNA are more stable than those of the cRNA (ΔG = -10.85 kcal/mol versus ΔG = -6.7 kcal/mol, respectively, Figure 3B). This result is consistent with the fact that vRNA is packaged into the virion, and the increased stability of this interaction would be necessary to protect the viral genome when the virions travel to infect other cells [7]. However, the structural differences observed between the models of NCRs from viruses with high and medium virulence, for both the vRNA and the cRNA, are not sufficient to attribute a potential role to the NCR as a virulence determinant. The RdRp capacity to efficiently bind to each secondary structure in the different isolates studied may constitute a potential virulence determinant; however, the binding efficiency remains to be elucidated because the complete sequences for the subunits of the polymerase (PA, PB1 and PB2) are not currently available, nor are their crystallographic structures. The lack of this information prevents the modeling of the interaction between these nucleotides and the binding or catalytic amino acids of the viral polymerase. Despite these limitations, this study shows that by using bioinformatic tools and a comparative approach, reliable models can be predicted for the secondary structures of the NCRs of ISAV. These models can be used to find key nucleotide positions involved in recognition by the RdRp (vRNA/cRNA) or the ribosome (mRNA) in other *Orthomyxovirus*es. This information allows the proposal of sites involved in the regulation of the initiation of different viral life cycle stages of ISAV at the levels of transcription, replication, and translation (Figure 4).

## Conclusion

The information obtained in this work allowed us to propose previously unknown sites that are involved in the regulation of different stages of the viral cycle, identifying new viral targets that may assist the development of future antiviral anti-sense strategies.

## Methods

### Sequences

Nucleotide sequences were compiled from the NCRs of eight genomic segments of six viral isolates of ISAV belonging to both the European (E) and North American (NA) genotypes. The isolates used in this study were ADL-PM 3205 ISAV07 (Isolate AD, E genotype), NBISA01 (Isolate NB, NA genotype) [20], 390/98 (Isolate 39, E genotype), 982/08 (Isolate 98, E genotype), Glesvaer/2/90 (Isolate GL, E genotype), and RPC NB 98/049 (Isolate RP, NA genotype) [21]. To date, these sequences correspond to all of the available information covering sequenced NCRs. No partial sequences are available.

### Sequence analysis: identification of conserved regions and consensus sequence

To define the boundary between the NCR and the respective viral ORF, a search for the start codon of each gene segment was conducted (Additional file 1: Figure S1). The vRNA and cRNA NCR sequences were then aligned using ClustalOmega program (http://www.clustal.org/omega/), locARNA (http://rna.informatik.uni-freiburg.de/LocARNA) and MAFT (http://mafft.cbrc.jp/alignment/server/) to determine conserved regions between segments and isolates. All three programs were run with default settings. A consensus sequence was generated for the NCRs of the genus Isavirus based on the consensus sequence of the influenza A virus [22] as a model to search for conserved genomic regions at the intergenus level.

### Description of the expression of gene regulatory element

A search was performed to discover the signals responsible for the regulation of different stages of the viral life cycle. To determine sequences related to the initiation of transcription, all nucleotides that were described in the influenza A vRNA as binding sites for the viral RNA polymerase determined using a bibliographic search [8,9,15,35] were used to propose potential candidates for regulating this interaction in ISAV. Concurrently, 2D modeling of the interaction of the NCRs of the vRNA was performed to analyze the formation of structural domains similar to those described in the influenza vRNA. This analysis was performed for each genome segment of the six available ISAV isolates. To this end, four computer programs that minimized free energy were used: RNAfold (http://rna.tbi.univie.ac.at/cgi-bin/RNAfold.cgi), RNAalifold (http://rna.tbi.univie.ac.at/cgi-bin/RNAalifold.cgi), RNAstructure (http://rna.urmc.rochester.edu/RNAstructureWeb/), Sfold (http://sfold.wadsworth.org/cgi-bin/srna.pl), locARNA (http://rna.informatik.uni-freiburg.de/LocARNA) and Mfold (http://mfold.rna.albany.edu/?q = mfold/RNA-Folding-Form). For all programs, 15 °C was used as the temperature setting. In parallel, the folding of the NCR of segment eight was modeled in 3D using the program RNAcomposer (http://rnacomposer.cs.put.poznan.pl) to visualize the spatial distribution of the molecular architecture observed in the 2D models. To determine sequences related to the initiation of replication, an analysis was conducted similar to the analysis mentioned above. However, the analysis was modified so that the cRNA of the viral segments was used as the template to search for nucleotide candidates that interact with the viral RNA polymerase [15] and for 2D and 3D modeling.

A separate search strategy was used to identify sites that regulate viral translation. The 5′ NCR end of the mRNA was analyzed for the degree of similarity to the Kozak consensus sequence (GCC^A^/_G_CCAUGG), in which the A of the AUG corresponds to the +1 position [23,24] of the AUG start codon of each viral protein for each isolate. An arbitrary ranking of the strength of the AUG as a Kozak sequence was assigned according to the presence or absence of two nucleotides critical for efficient translation initiation, A/G^-3^ and A^+4^[23], and intermediate values 0 to +5 were included based on the presence or absence of the remaining nucleotides in the Kozak consensus determined in this study. Using these data, a score of the strength of the genomic context for the AUGs in the segments of the studied isolates (according to [36]) was generated. If the two critical nucleotides were present surrounding the AUG, then this AUG was classified as optimal for translation initiation; when only one matched, the AUG was considered a strong candidate for translation initiation; if there was no match with the consensus sequence, then the AUG was classified as a weak candidate for translation initiation. To determine the signals involved in the termination of transcription and the polyadenylation signal, a U stretch was identified in the 3′ end of the NCR by homology to influenza based on previously described data [4,16].

## Competing interests

The authors declare that they have no competing interests.

## Authors’ contributions

AD conceived the study; AD and KG prepared the experiments, participated in their design and drafted the manuscript; AN, GH commented on the manuscript; JR was involved in the critical revision of the manuscript for important intellectual content. All authors read and approved the final manuscript.

## Supplementary Material

Additional file 1: Figure S1 Alignments of the 3′ and 5′ NCR in the vRNA of the different isolates. The nucleotide residues labeled as N represent the ORF for each segment. Click here for file

Additional file 2: Figure S2 Predicted secondary structure from the RNAfold program of the sequences of the 5′ and 3′ termini in the vRNA of each genomic segment. Free energies (ΔG) are reported for each structure. The ORFs present in each segment are represented by “X”. Although differences at the nucleotide level are reported among the isolates for segments 1, 4, 6, and 8, these differences do not affect the predicted folding structure. Click here for file

Additional file 3: Figure S3 Predicted secondary structure from the RNAfold program for the sequences of the 5′ and 3′ termini in the cRNA of each genomic segment. Free energies (ΔG) are reported for each structure. The ORFs present in each segment are represented by “X”. Although differences are reported at the nucleotide level among the isolates for segments 1, 4, 6, and 8, folding is only affected in segments 4 and 6 (only one model is shown). Click here for file
