# Supplementary material for: Virtual screening of gene expression regulatory sites in non-coding regions of the infectious salmon anemia virus
Source: BMC Res Notes. 2014 Jul 28;7:477. doi: 10.1186/1756-0500-7-477 (PMC4132239; doi:10.1186/1756-0500-7-477)
Supplement: Additional file 1: Figure S1 — Alignments of the 3′ and 5′ NCR in the vRNA of the different isolates. The nucleotide residues labeled as N represent the ORF for each segment. [file 1756-0500-7-477-S1.doc]

Additional file 1: Figure1S
