# Supplementary figures and images for: Virtual screening of gene expression regulatory sites in non-coding regions of the infectious salmon anemia virus
Source: BMC Res Notes. 2014 Jul 28;7:477. doi: 10.1186/1756-0500-7-477 (PMC4132239; doi:10.1186/1756-0500-7-477)

Additional file 2: Figure S2


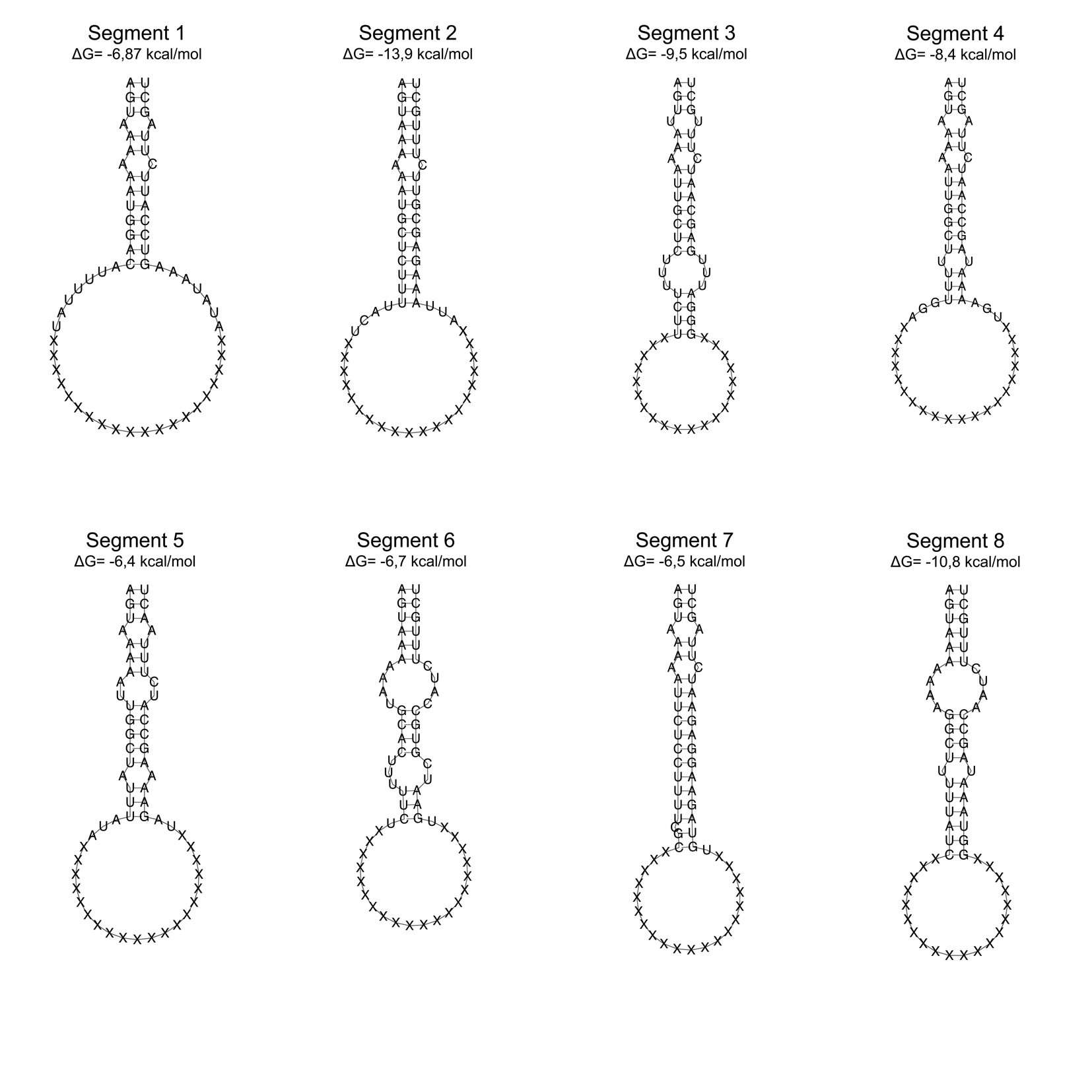

Supplement: Additional file 2: Figure S2 — Predicted secondary structure from the RNAfold program of the sequences of the 5′ and 3′ termini in the vRNA of each genomic segment. Free energies (ΔG) are reported for each structure. The ORFs present in each segment are represented by “X”. Although differences at the nucleotide level are reported among the isolates for segments 1, 4, 6, and 8, these differences do not affect the predicted folding structure. [file 1756-0500-7-477-S2.doc]

Additional file 3: Figure S3


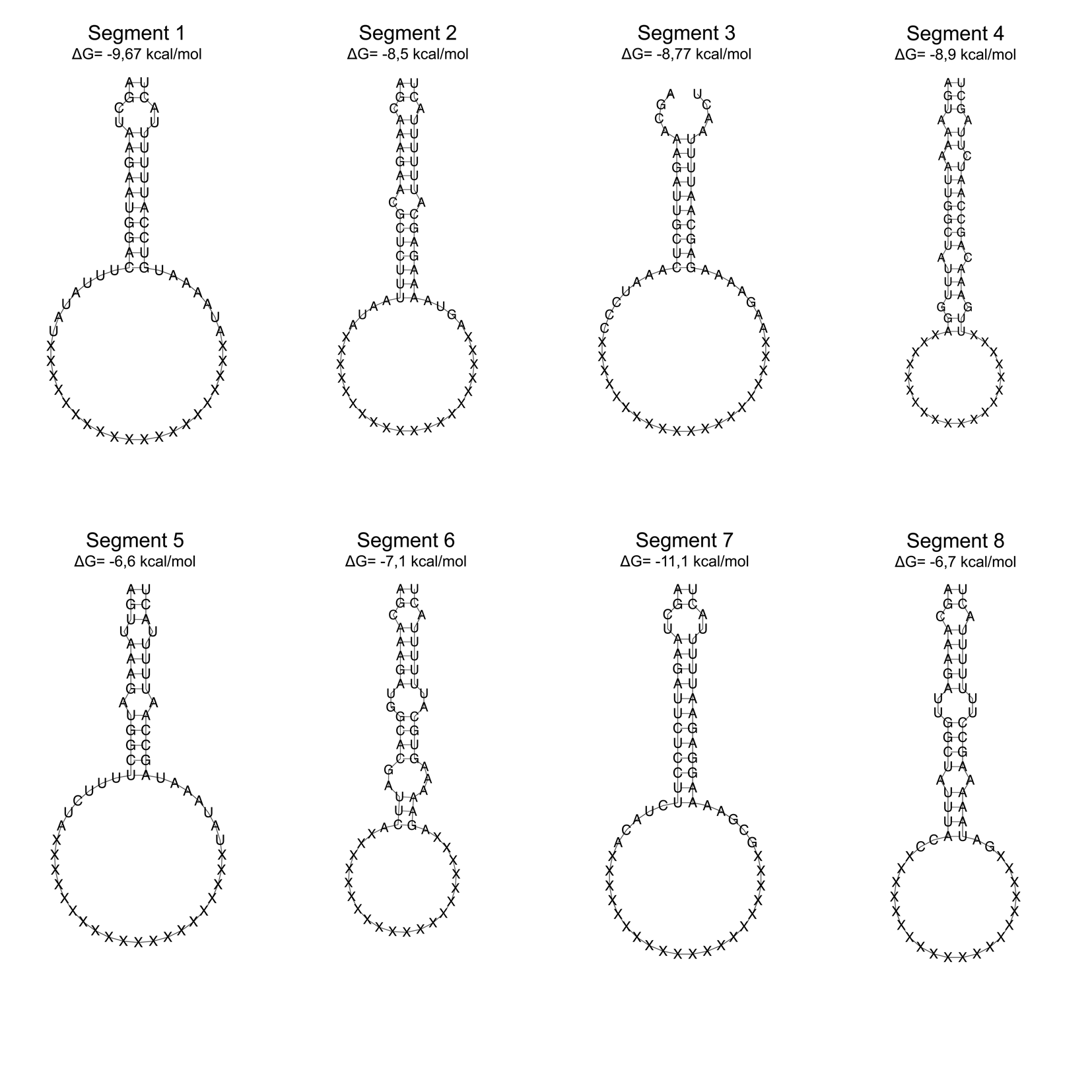

Supplement: Additional file 3: Figure S3 — Predicted secondary structure from the RNAfold program for the sequences of the 5′ and 3′ termini in the cRNA of each genomic segment. Free energies (ΔG) are reported for each structure. The ORFs present in each segment are represented by “X”. Although differences are reported at the nucleotide level among the isolates for segments 1, 4, 6, and 8, folding is only affected in segments 4 and 6 (only one model is shown). [file 1756-0500-7-477-S3.doc]
